# TECHNOLOGY-BASED INNOVATIONS IN LONG-TERM CARE HOMES DURING COVID-19 AND RELATED INCLUSION PRACTICES

**DOI:** 10.1093/geroni/igae098.2162

**Published:** 2024-12-31

**Authors:** Charlene Chu, Sumaya Bhatti, Yanjun Duan, Suzanne Santos, Michael Lepore, Ruth Melo, Franziska Zuniga, Lisa Cranley

**Affiliations:** University of Toronto, Toronto, Ontario, Canada; University of Toronto, Toronto, Ontario, Canada; University of Toronto, Toronto, Ontario, Canada; University of São Paulo, São Paulo, Sao Paulo, Brazil; University of Massachusetts Amherst, Amherst, Massachusetts, United States; University of São Paulo, São Paulo, Sao Paulo, Brazil; University of Basel, Basel, Basel-Stadt, Switzerland; University of Toronto, Toronto, Ontario, Canada

## Abstract

The COVID-19 pandemic has magnified challenges in Long-Term Care Homes (LTCHs), driving the adoption of technology-based solutions. The scoping review to explore technology-based innovations into a LTCH during COVID-19 in Canada, the US, Brazil and Switzerland produced 60 studies. The most reported technologies were use of telemedicine/virtual medical care tools that allowed for remote delivery of health care (n=16) within LTCHs, and robotics for clinical tasks and social interaction (n=14). Less frequently reported technologies were: wearables for health monitoring (n=2), mobile apps to improve residents’ mental well-being (n=2), and call light systems (n=1) between residents and staff. Most technologies were introduced by researchers (n=38) which primarily engaged with staff (n=30) and residents (n=24). Studies with telemedicine and robotics (n=30) focused on exploring the feasibility and acceptability of technology with the aim to improve optimal resident care and increase social connectedness. Notably, the most common goals of the technology-based innovations were to provide virtual medical care and to engage residents socially. The majority of the papers had active engagement (n=24) which relies on stakeholder’s actions (i.e. video calls). We also examined the level of inclusion and engagement of residents, their families, and staff and found varying levels of engagement. the term “co-design” was explicitly used (n=7) where the innovation was designed together with the stakeholders (for example, stakeholder opinions were considered during the process). This paper describes the engagement of stakeholders of these technological innovations, and points to the need for future research in this area.

